# Study on the fracture propagation of ground fissures with syn-depositional structure in Fenwei Basin, China

**DOI:** 10.1038/s41598-024-61657-4

**Published:** 2024-05-13

**Authors:** Quanzhong Lu, Cong Li, Rendao Liu, Yuemin Sun, Xinyu Mao, Feilong Chen

**Affiliations:** 1 School of Geological Engineering and Geomatics, Chang’an University, Xi’an, 710054 Shaanxi China; 2 Key Laboratory of Earth Fissures Geological Disaster, Ministry of Natural Resources, Xi’an, 710054 Shaanxi China; 3 Observation and Research Station of Ground Fissure and Land Subsidence, Ministry of Natural Resources, Xi’an, 710054 Shaanxi China

**Keywords:** Fenwei Basin, Syn-depositional ground fissure, Physical simulation test, Fracture propagation, Natural hazards, Geology, Sedimentology, Tectonics

## Abstract

In Fenwei Basin, most of the tectonic ground fissures show characteristics of growth faults on the section. They continue to destroy the engineering properties of soil at different depths. This has introduced significant security risks to the construction processes of deep underground spaces. However, there are few studies have been conducted on syn-depositional ground fissures. Therefore, in this study, a physical simulation test was used to study the fracture propagation of syn-depositional ground fissures. The characteristics of sections and surface fractures were analyzed. The engineering properties of model soil were divided into bad and poor areas. The syn-depositional ground fissure fracture propagation process was divided into five phases. The results show that soil profile exhibited a composite Y-shaped fracture morphology. Syn-deposition affects the fracture angle and healing state of fractures. The soil strain and surface displacement were positively correlated with the number of deposition layers. The conclusions of this study provide a theoretical geological basis and practical engineering significance for design of deep underground space structures.

## Introduction

**Figure 1:**
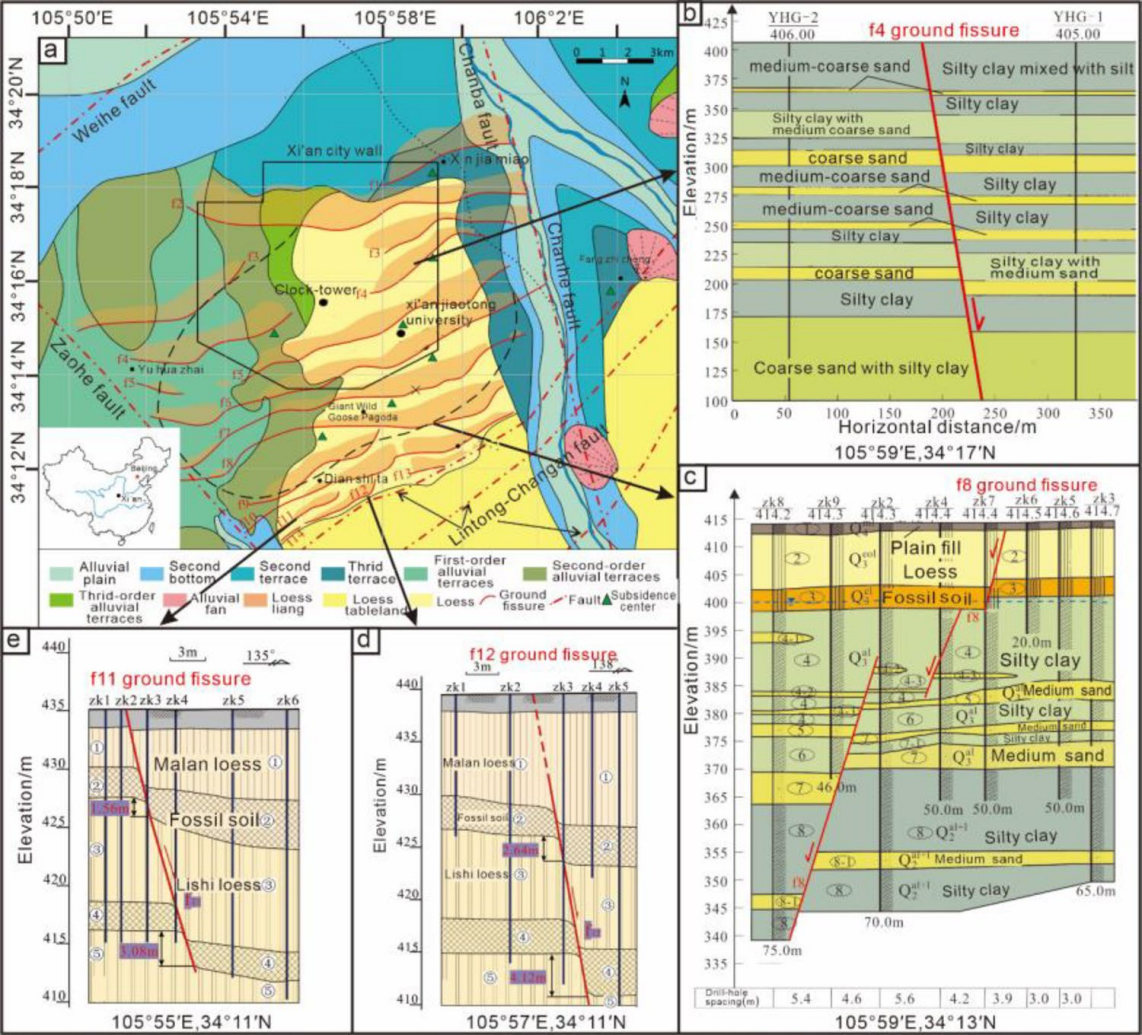
Distribution of ground fissures in Xi'an and sections of some ground fissures. (**a**) Distribution of ground fissures in Xi'an^9^. (**b**–**e**) f4, f8, f12, and f11 stratigraphic sections.

Ground fissures are geological disasters caused by surface deformations or ruptures^1–6^. In the Fenwei Basin, the Xi 'an area has the largest number of ground fissures and is also the most representative. In Xi'an, 14 ground fissures have been found (Fig. 1a), with an extension length of more than 200 km^7^. This poses a significant threat to public infrastructure and the safety of human lives and property.

The majority of ground fissure disasters stem from the "resurrection" or reactivation of hidden ground fissure under overburden, triggered by external geological forces like tectonic activity, loess wetting, surface water seepage, groundwater extraction, or heavy rainfall. These fractures then rupture and extend to the surface, causing significant damage to structures. For instance, the Feng village fracture in Dali County, Shaanxi Province, resulted from combined extension and fracture activities in the Weihe River Basin, leading to surface misalignment and fracture formation^8^. Similarly, fractures in Weizhuang and Xibai villages in Dali County, Shaanxi Province, were caused by loess wet subsidence and surface water seepage due to agricultural irrigation, resulting in surface exposure of the fractures^3,8^.

There are many ways to study ground fissure hazards. Remote sensing technology, geophysical exploration and field investigation methods can analyze the distribution, development and activity characteristics of ground fissures^10–13^. Compared to other methods, physical model tests exhibit different advantages in studying the ground fissure extension process. This method can visually monitor the gradual process from the beginning to the end of the disaster by simulating actual geological movements and human engineering activities. The physical simulation experiment can study the process of extensional structure in the basin^14–17^. Some scholars used physical model tests to analyze the influencing factors of the fracture propagation process of concealed ground fissures^18–20^.

Some scholars investigated the effects of soil cementation properties, burial depth of pre-existing fracture, relative densities of overlying sand layers, fault dip angles, changes in soil properties, rupture extension, minimum fault dislocation for surface extension, and the relationship between surface deformation zone width and soil layer thickness^19,21–23^. Other scholars studied rainfall-induced ground fracture reactivation, where surface water infiltration into fracture zones affects the lower non-fractured zone^3,24^.

These scholars primarily focus on soil properties, thickness, pre-existing fractures, and rainfall impact on hidden ground fissure propagation, emphasizing shallow surface fracture characteristics, their influence range, and validating the ground fissure genesis mechanism. In contrast, ground fissure development in the Xi'an area is largely influenced by tectonics, as evidenced by "growth faults" in cross-sections, indicating a close link to syn-sedimentation in tectonic ground fissure formation (Fig. 1b–e). And, each deposition process adjusts the stresses and strains overlying the fracture. This will have an effect on the expand path of the old fracture.

The development of ground fissures in Xi 'an is mainly affected by tectonic action^12,25–27^.

Therefore, in this study, we used a large-scale physical simulation test to study the fracture propagation of syn-depositional ground fissures. The tests used multiple layer laydowns to simulate the process of layer syn-sedimentation in the natural state. We analyzed the fracture of the soil profile, surface deformation, and the range of the influence zone. Here, we summarize the developmental stages of fracture propagation. These research results can explain the formation of syn-depositional ground fissures. And providing a theoretical basis and scientific basis for urban construction and disaster prevention in Xi'an.

## Physical model test

### Test design

**Figure 2:**
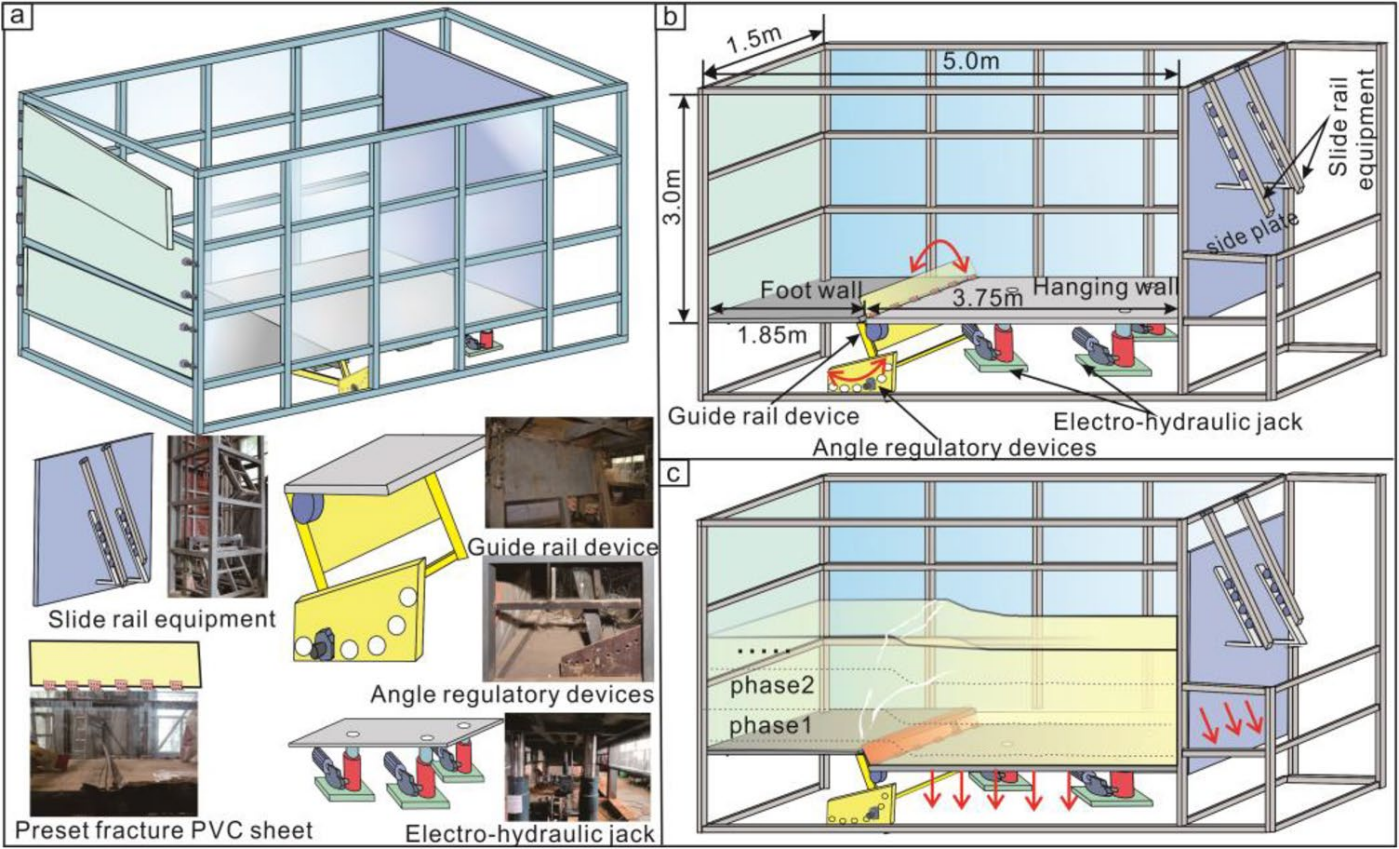
Model box diagram. (**a**) The physical map of each part of model box. (**b**) Box structure and size. (**c**) Demonstration of model box test process.

The ground fissures in Xi'an are mainly vertical differential settlement, and the dip angle of deep fissures is 70°–90°. The model box shown in Fig. 2 was used for testing. The size of the model box is 5.0 m × 1.5 m × 3.0 m. The test simulated a normal-fault ground fissure with a length of 50 m vertical to the ground fissure and a width of 15 m along the ground fissure. The preset fracture dip angle was set to 75°.

The maximum activity rate of Xi'an ground fissures is about 56 mm/a^7,9,28^. In this study, the ground fissure activity and multiple sedimentary processes of the layers were simulated. We set the preset fracture activity rate to 0.5 mm/s. And, We divided the test into five times, each activity for 60 s.

### Test platform

The equipment comprised a stainless-steel box, slide rail, and hydraulic jacks. The bottom of box is spliced by steel plates with dimensions of 1.85 m × 1.5 m × 0.03 m and 3.15 m × 1.5 m × 0.03 m. They divided the model box into a footwall and hanging wall of model layer. The PVC plates were installed at the top of the guide rail, in contact with hanging wall steel plate. This was considered to be a pre-existing fracture.

The model box is composed of tempered Plexiglas, formwork, and stainless steel plates, with significantly greater stiffness than the modeled soil. It is assumed that material stiffness does not affect the test, and friction and shear forces between the soil and the box are negligible. Thus, the model soil experiences only gravity and interparticle cohesion.

## Model procedure

### Model layer preparation

**Figure 3:**
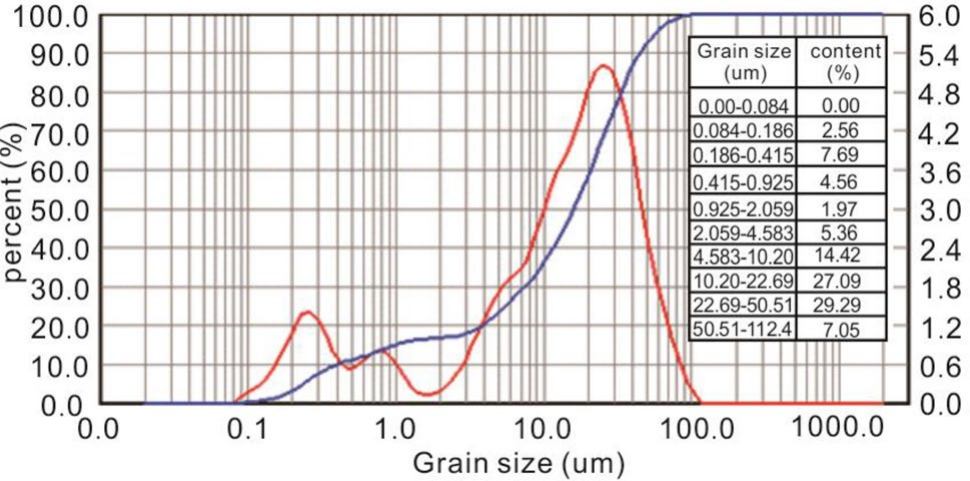
Laser particle size analysis of model soil.

The Q3 Malan loess from Xi'an, Shaanxi Province was chosen as the model soil material for the test. The undisturbed soil samples of loess had a density ranging from 1.75 to 1.95 g/cm^3^ and a moisture content between 19 and 22%^29^. Prior to the test, workers sieved the soil to remove debris and larger pieces using a 2-mm sieve to ensure material homogeneity (Fig. 3). To achieve similar water content and density as the original soil, the screened soil was moistened and left for several days. Subsequent tamping tests were performed to determine the density of the model soil, and the physical and mechanical parameters were obtained through direct shear testing (Table 1).

**Table 1:** Physical and mechanical parameters of modeled soils.

Parameters	Density g/cm^3^	Water content/%	Cohesion/kPa	Internal friction angle
Model soil	1.61	22.1	40.72	15.39

### Test procedure

**Figure 4:**
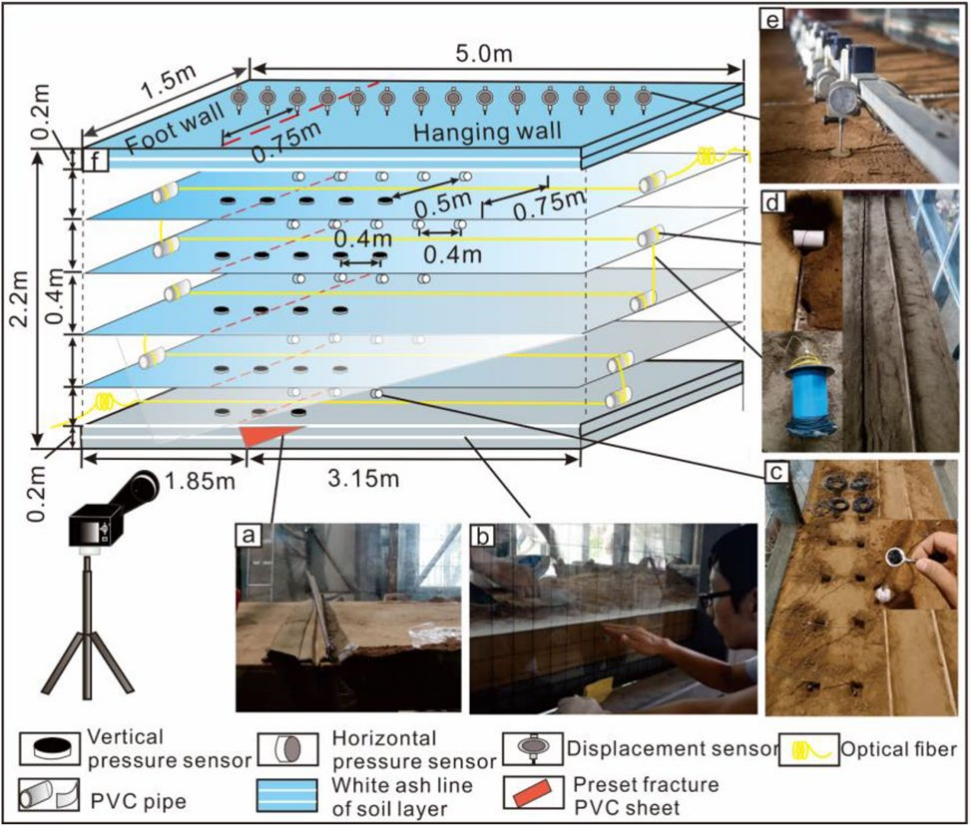
Monitoring equipment layout diagram. (**a**) Preset fractured pvc sheets. (**b**) White ash line of soil. (**c**) Soil pressure monitoring equipment. (**d**) Optical fiber embedding process. (**e**) Surface displacement monitoring equipment. (**f**) Buried location diagram of various monitoring components. The bottom plate of the model box was 0 m thick, and the surface of the soil was 2.2 m thick.

A total of five hanging wall settlement simulations (Phase 1 to phase 5) were performed for the test. Phase 1: The angle of the preset concealed fracture plate to the guide rail was set to 75° in the first step (Fig. 4a). A 0.2 m thick layer was laid to secure the preset fracture plate (Fig. 4b), with sensors and optical fibers buried in this layer (Fig. 4c,d). Subsequently, a 0.4 m thick layer was added, and a vertical linear displacement meter was installed on the surface (Fig. 4e). In the third step, following completion of stratigraphic modeling, the electric jack under the bottom plate of the model box was activated, settling the upper plate for 60 s at a rate of 0.5 mm/s. Concurrently, a digital camera captured a rupture shot of the profile, and pressure and displacement data were collected.

Phase 2: After the hanging wall of the model layer in phase 1 settled for 60 s, a 0.2 m thick soil layer was placed atop it, embedding soil pressure sensors. This was followed by another 0.2 m thick soil layer and installation of a vertical linear displacement meter. Data acquisition followed phase 1 procedures. Phases 3–5 replicated the modeling steps of the phase 2. In each phase, a 0.2 m thick soil layer was added above the previous stratum, with soil sensors buried. Then a 0.2 m thick layer of soil was continued and a vertical linear displacement meter was installed at the surface.

## Results

### Fracture propagation analysis of model section

**Figure 5:**
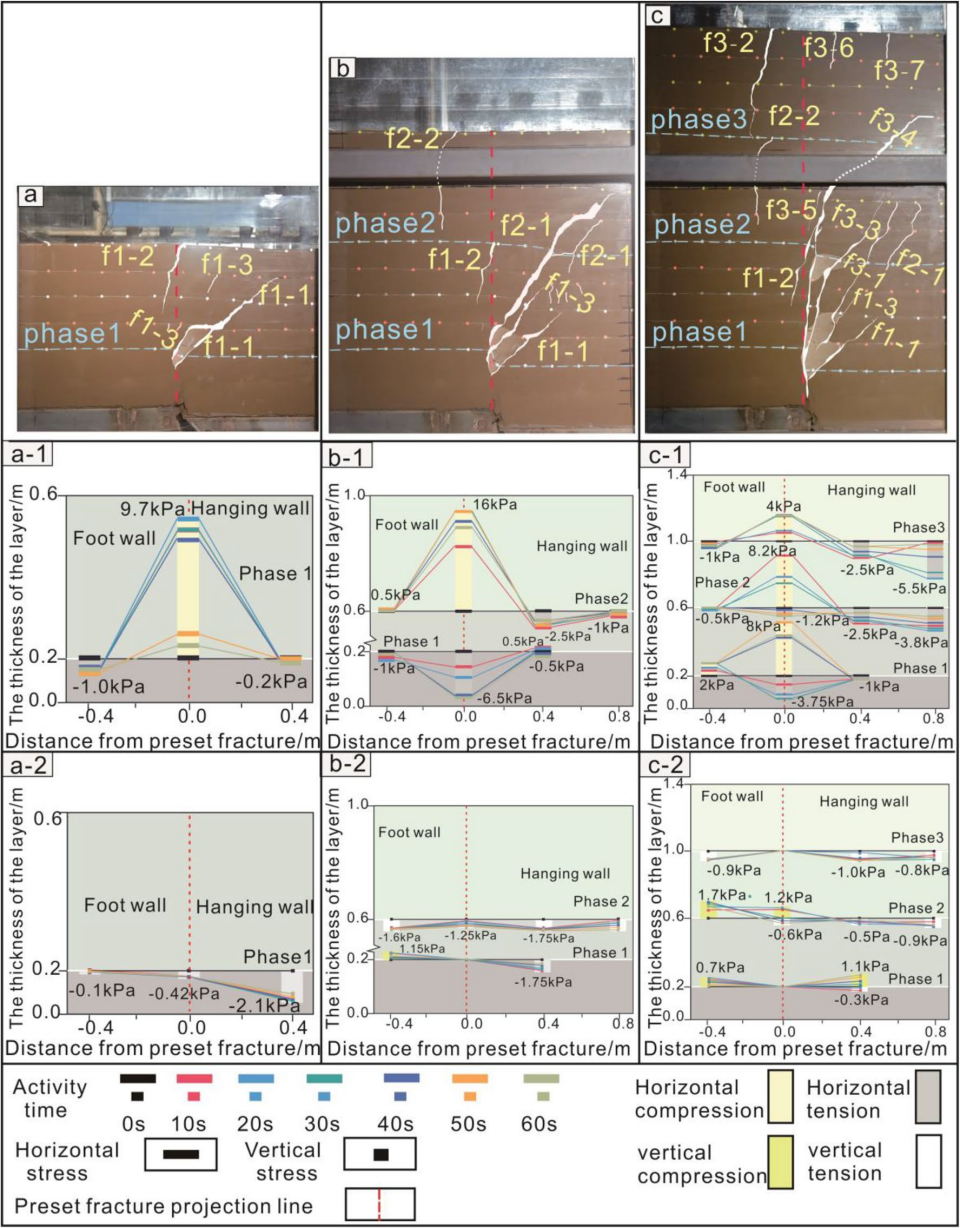
Soil section and soil pressure of the 1st–3rd phase test. (**a**–**c**) The 1st–3rd phase section fracture. (a-1, b-1, and c-1) The 1st–3rd phase horizontal stress. (a-2, b-2, and c-2) The 1st–3rd phase vertical stress. Horizontal distance: the preset fracture position is 0 points, the hanging wall is positive, and the footwall is negative. Vertical distance: the bottom plate of the footwall is 0 points, and the distance toward the top of soil layer is positive. Fracture f1-1: the first fracture in process of the first phase. Soil pressure: The compressive stress is positive, and the tension stress is negative.

**Figure 6:**
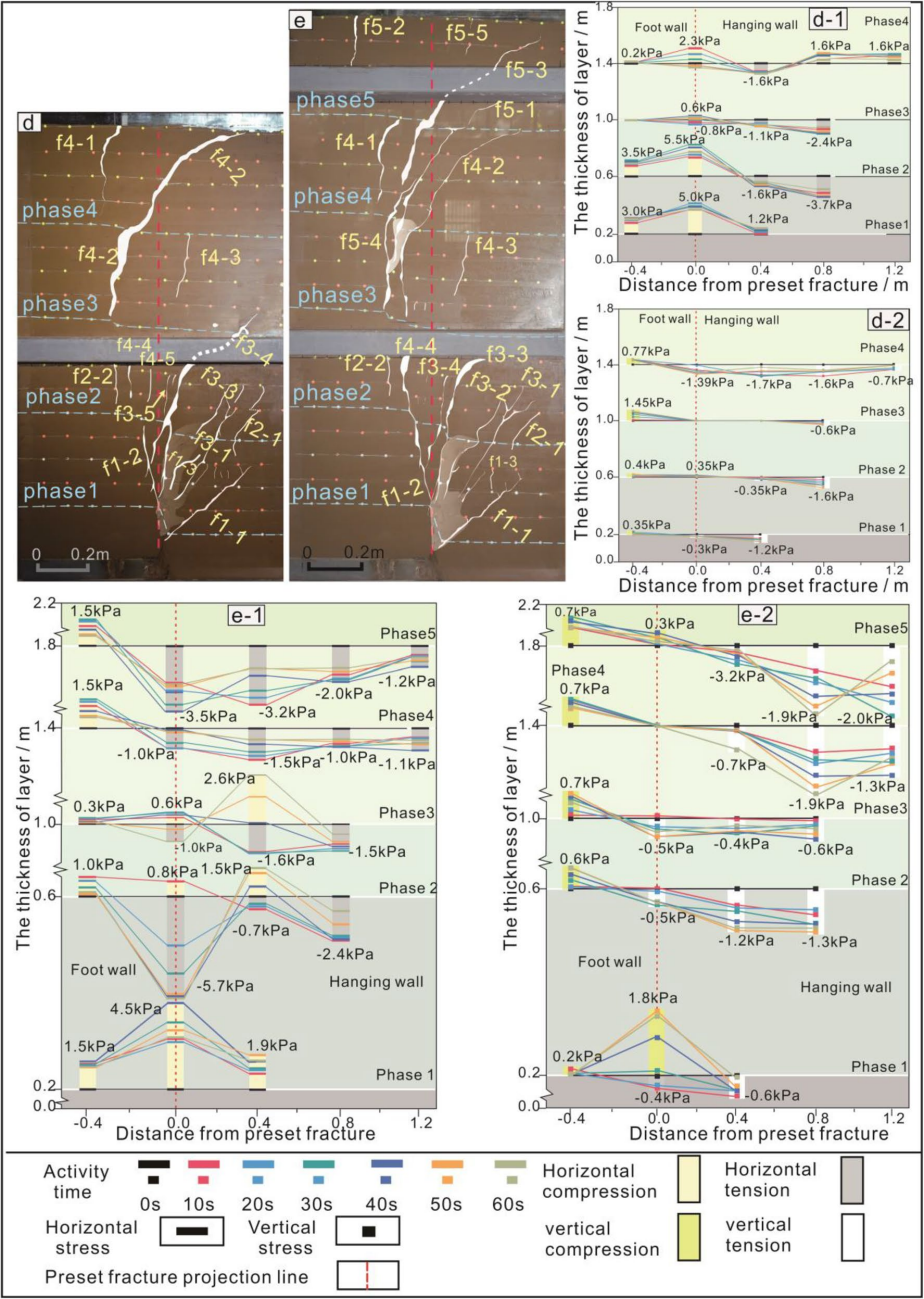
Soil section and soil pressure of the 4th–5th phase test. (**d** and **e**) The 4th–5th phase section fracture. (d-1 and e-1) The 4th–5th phase horizontal stress. (d-2 and e-2) The 4th–5th phase vertical stress.

The fracture of model section gradually changed from 'Y' type (Fig. 5a–c) to composite 'Y' type (Fig. 6d,e). This section presents a typical syn-depositional structure. The main anti-dip fractures extend from the tip of the preset fracture to surface by f1-2, f4-4, f5-4, and f5-3. Secondary anti-dip fractures (f2-1, f3-1, f4-3, etc.) mainly developed at the bottom of the hanging wall layer. However, they did not continue to propagate after the third phase.

The main anti-dip fracture morphology was nearly 45° oblique line during the first phase and second phase. It then transformed into a 'rickets' shape with a nearly upright bottom and a curved top. It gradually moved closer to the footwall, and there was a 'traceability' performance. This was because the stress state of bottom soil layer changed.

In the first phase, the bottom and middle soil layers were primarily subjected to horizontal compression accompanied by vertical tension, forming the main anti-dip fractures (Fig. *5a-1,a-2). In the second-third phase, the fracture continued to expand. The hanging wall is continuously subjected to horizontal tensile stress (Fig. 5b-1, b-2, c-1, c-2). In the fourth phase, the range of tensile stresses in the soil layer gradually increases (Fig. 6d-1, d-2). In the fifth phase, the main anti-dip fractures in the bottom soil layer were affected by horizontal and vertical pressures and shear force. Therefore, the shapes of fractures in this area changed (Fig. 6e-1,e-2). Under the combined action of horizontal and vertical tensile stresses in the middle soil layer, the existing fractures restarted 'resurrection' as a starting point to begin to expand (Fig. 6e-1,e-2).

### Model trench section analysis

**Figure 7:**
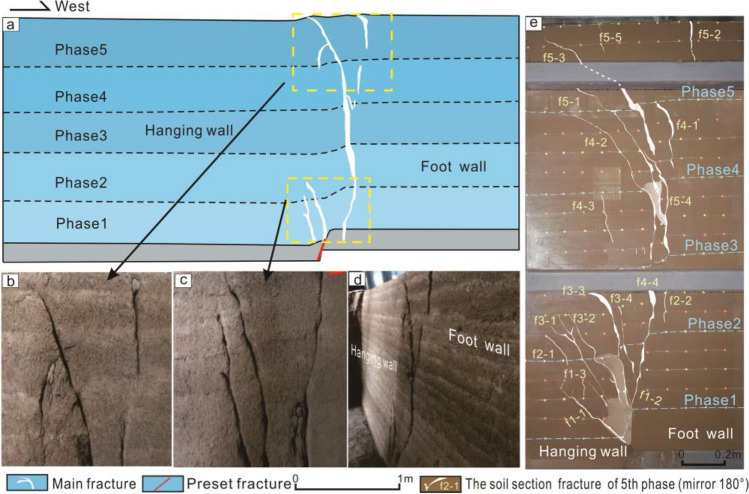
Soil section and trench section. (**a**) Soil trench section. (**b**) The main fracture of soil layer at the top of trench section. (**c**) The main fracture of soil layer at preset fracture of trench section. (**d**) Photograph of trench profile. (**e**) The 4th–5th phase section fracture.

The dip angle of main anti-dip fracture changed to nearly 90° (Fig. 7a,b). Secondary anti-dip fractures f3-3 and f1-1 were observed at the tip of preset fracture (Fig. 7c) and main anti-dip fractures f5-3, f5-4, f4-4, and f1-2 throughout layers in the model trench section (Fig. 7d). This is consistent with ground fissure trench section phenomenon caused by field structure. This indicates that the syn-depositional action gradually connected main anti-dip fractures in each phase and finally formed a single near-vertical main fracture.

Secondary anti-dip fractures (f2-1, f3-1, f3-2, f5-1, and f4-3) on hanging wall of the model soil section completely healed in model trench section (Fig. 7d,e). However, these healed anti-dip fractures were not observed in model, and soil is also damaged. In the future geological development period, these 'healed' fracture zones may rupture again. Therefore, in addition to avoiding main fracture zone during the construction of underground space projects, 'healed' secondary fracture zones should also be circumvented.

Analysis of Figs. 6e and 7 reveals that syn-sedimentary ground fissures in the layer progressively penetrate from the root of the main anti-dip fracture in each stage, forming primary fractures with angles of 80°-90°. Studies of fractures in the Fenwei Basin show similar single near-vertical fracture morphology as observed in the tests, with gentle dips at depth gradually increasing near the surface. While direct observation of different stages of surface fracture in the field is difficult, the main fractures in field layer exhibit a downward tendency similar to the tests, suggesting consistency between surface fractures in the field and test results, with surface deformation areas gradually shifting towards the footwall direction at each depositional stage.

### Analysis of surface displacement and soil strain

**Figure 8:**
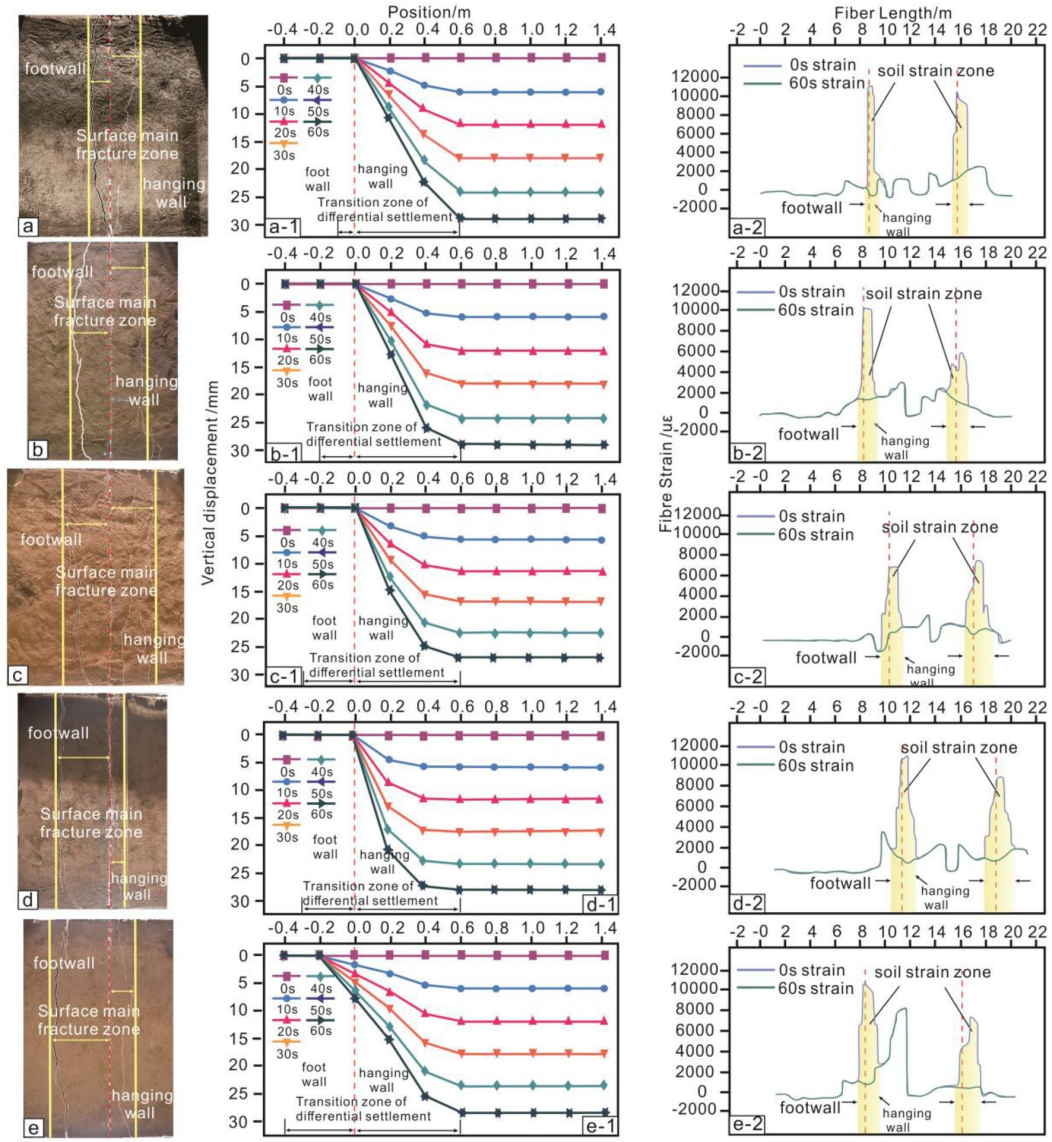
Surface fracture, surface displacement curve, and soil strain curve. (**a**–**e**) The surface fracture of 1st–5th phase. (a-1, b-1, c-1, d-1, and e-1) The surface displacement curve of 1st–5th phase. (a-2, b-2, c-2, d-2, and e-2) The soil strain curve of 1st–5th phase. The preset fracture position is X-coordinate zero point of surface subsidence curve. The direction toward hanging wall is positive, and the direction of footwall is negative. The fiber is tensile, and the strain is positive. The fiber is compressed, and the strain is negative. There are two optical fibers in buried position at each stage, so there will be two strain regions in the Figure.

From the first phase to the fifth phase, soil strain zone increased from 1.64 m (footwall 0.35, hanging wall 1.29 m) to 2.66 m (footwall 1.66 m, hanging wall 1.0 m) (Fig. 8a-2–e-2). The surface differential subsidence zone increased from 0.7 m (footwall) to 1.0 m (footwall 0.4 m, hanging wall 0.6 m) (Fig. 8a-1–e-2). This shows that the number of depositions has a positive correlation with soil strain and surface subsidence. Both regions gradually develop toward the footwall.

From the first phase to the fifth phase, the width of surface main rupture zone of the footwall gradually increased (Fig. 8a–e). However, in the fourth phase, the width of hanging wall was smaller than previous. This is not the same as development law of soil strain and surface differential settlement. The reason for this difference may be that the cohesion of soil can help fracture produce a wider gap. When the number of vertical dislocations in middle and bottom soil layers increased, narrow voids appeared in the lower part of hanging wall anti-dip fractures.

Surface subsidence in Xi'an strongly correlates with ground fissure orientation^30–32^. Differential settlement occurs at the surface due to ground fissure extension from deep fracture. In the fifth stage of the test, the maximum hanging wall displacement is 0.75 mm and the footwall is 28 mm (Fig. 8e-1). Comparatively, considering the middle section of the actual Xi'an f7 ground fissure over a year, its hanging wall settles by 23 mm, while the footwall settles by 1 mm^25^. This shows that the test results are close to the actual settlement. And this suggests that the model test can explain the differential settlement phenomenon presented by the real ground fissure at the surface.

### Quantitative analysis of fracture zone

From the first phase to fifth phase, width of the surface main rupture zone of footwall increased from 0.1 to 0.4 m, and hanging wall part was generally 1–4 times that of the footwall part (Table 2). In contrast, the proportion of soil strain zone in footwall increases from 25 to 30%. In addition, the deformation angle of footwall part of soil strain zone was between 55° and 75°. The deformation angle of footwall part of model section's main rupture zone is between 75° and 82° (Table 2). The anti-dip fracture moving to footwall does not change the trend in which deformation angle of footwall increases with increasing deposition.

**Table 2:** Quantitative statistical table of fracture zone.

Deposition phase	Layer thickness/m	Vertical activity of preset fracture/mm	Surface main fracture zone/m		Micro-deformation zone/m		Deformation angle of footwall	
			Hanging wall	Foot wall	Hanging wall	Foot wall	Section main fracture zone	Micro-deformation zone
the first phase	0.60	30.00	0.40	0.10	0.89	0.25	75°	55°
the second phase	1.00	60.00	0.40	0.20	1.22	0.35	75°	58°
the third phase	1.40	90.00	0.40	0.30	1.38	0.49	78°	65°
the fourth phase	1.80	120.00	0.40	0.30	1.70	0.72	80°	65°
the fifth phase	2.20	150.00	0.40	0.40	0.60	1.26	82°	75°

**Figure 9:**
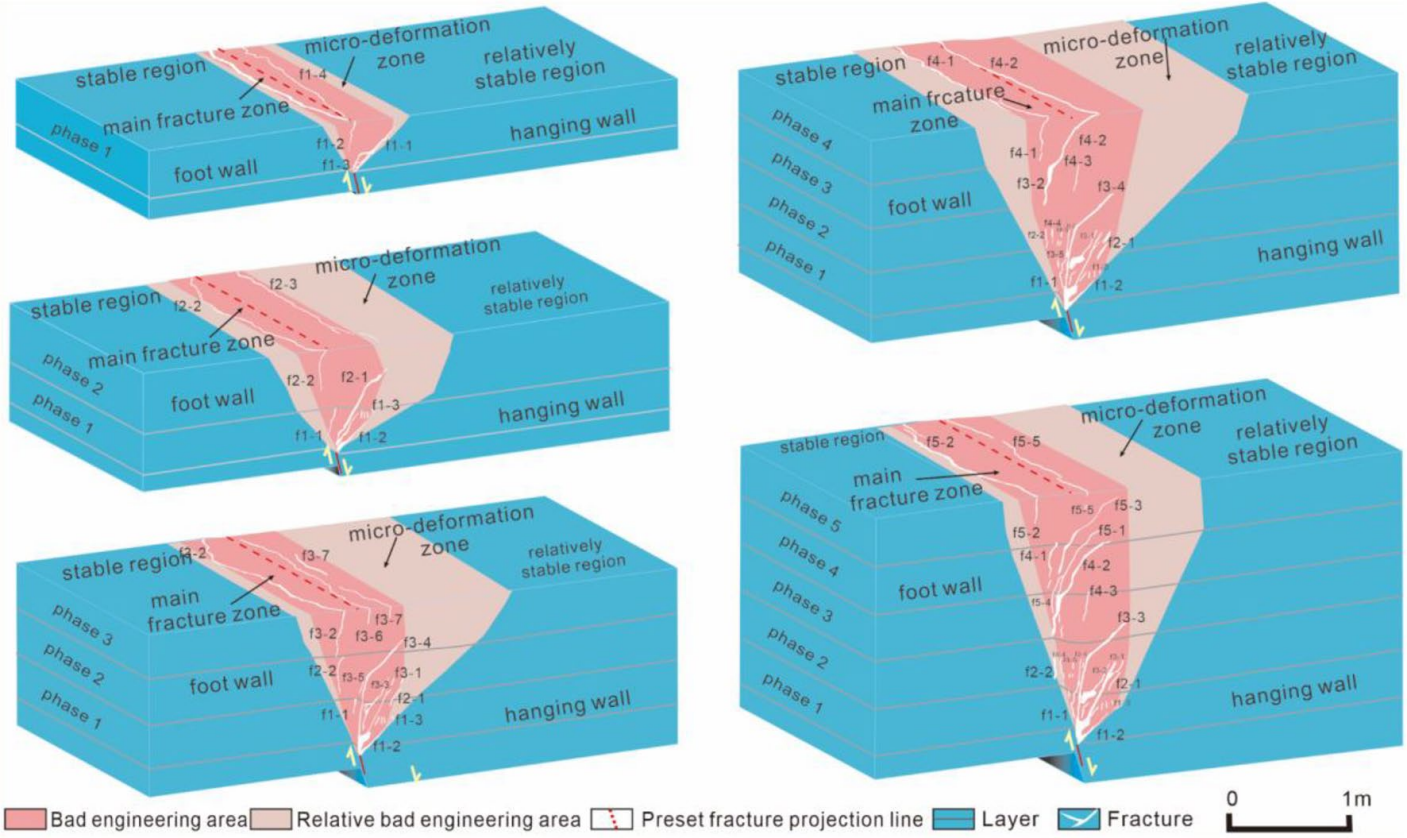
The model rupture partition.

The model section and surface where fractures appeared were main rupture zones (Fig. 9). The two areas where soil was broken could no longer carry out engineering construction. Therefore, these two areas are considered poor engineering areas.

Beyond these two areas, there was a micro-deformation area (Fig. 9). Although soil was not destroyed, soil strain was extremely high. This indicated that soil in this area was disturbed. Therefore, micro-deformation area was set as the worst engineering area. Therefore, these areas should be avoided in engineering construction projects.

The surface deformation area (Fig. 9) increases with layer thickness. And the deformation trend shifts towards the footwall, aligning with existing studies^26,27,33^. Notably, in phase 4, hanging wall layer deform over 2.1 m, while footwall deform over 1.02 m (surface main fracture zone and micro-deformation zone from Table 2). With a 5 m model box simulating a 50 m strike length, the test represents 21 m hanging wall deformation and 10.2 m footwall deformation, meeting Xi'an ground fissure investigation regulations (DBJ61T182-2021) of 20 m and 12 m, respectively. This demonstrates alignment between test results and regulatory standards.

The deformation angle is defined as the angle between preset fracture tip and profile fracture zone. Because engineering construction is usually conducted on a footwall, the avoidance distance of footwall is generally considered. In this study, the deformation angle of hanging wall was not considered.

## Discussion

### Fracture rupture expansion stage

**Figure 10:**
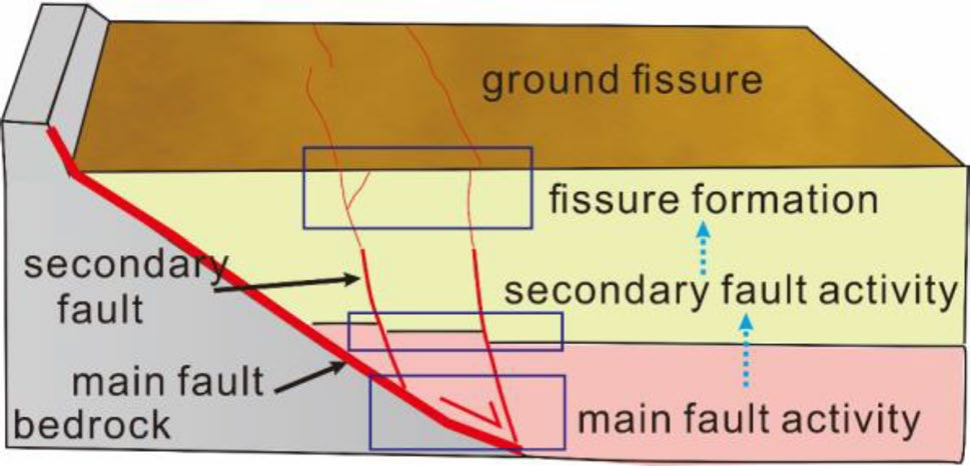
The development phase fissures in Fenwei Basin^36^.

The tectonic ground fissures in Fenwei Basin are primarily controlled by active faults. Ground fissures are densely distributed along fault zones in basin and are manifestations of surface fault activity. They are directly or indirectly connected to underlying faults^34,35^. The formation of ground fissures originating from hanging walls of faults in Fenwei Basin can be divided into three stages (Fig. 10): main fault activity, secondary fault activity, and fracture formation phase^36^.

**Figure 11:**
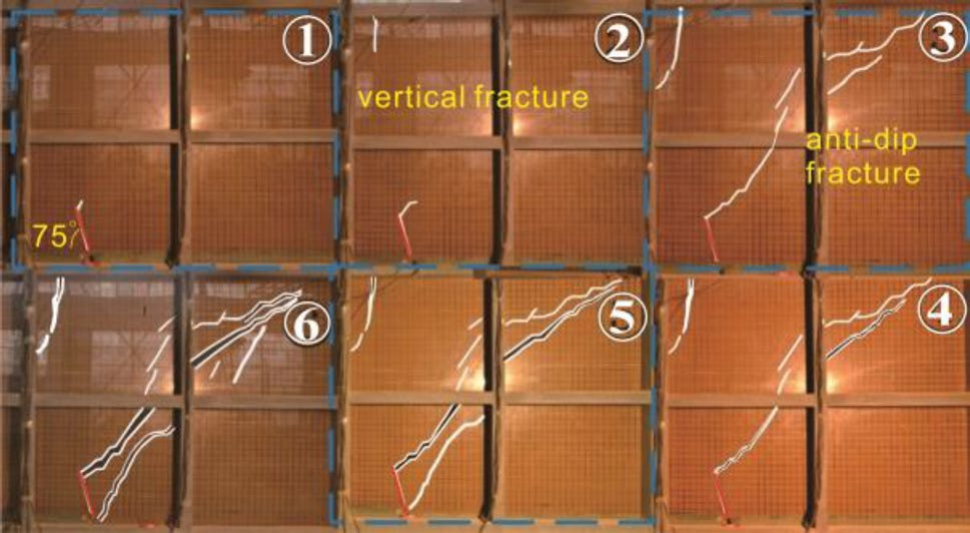
Buried ground fissure fracture propagation phase.

The fracture formation stage was similar to test process of fracture propagation of buried ground fissure (Fig. 11). This indicates that the long-term creep of the fault causes the near-surface loose rock and soil to break, which promotes the exposure and expansion of ground fissures. However, the study of buried ground fissures only uses the factors of single tectonic movement to explain the rupture process. The influence of continuous deposition of layer on pre-existing fractures and new fracture paths is not considered.

According to Fig. 7c, two fractures (f1-1 and f3-1) at the tip of preset fracture are also similar to the secondary faults of basin in profile structure. A preset fracture is regarded as main fault. Combined with the results of this test, we can infer that, after main fault activity stage, the first main anti-dip fractures gradually transformed into secondary faults in deep layer under sedimentation.

**Figure 12:**
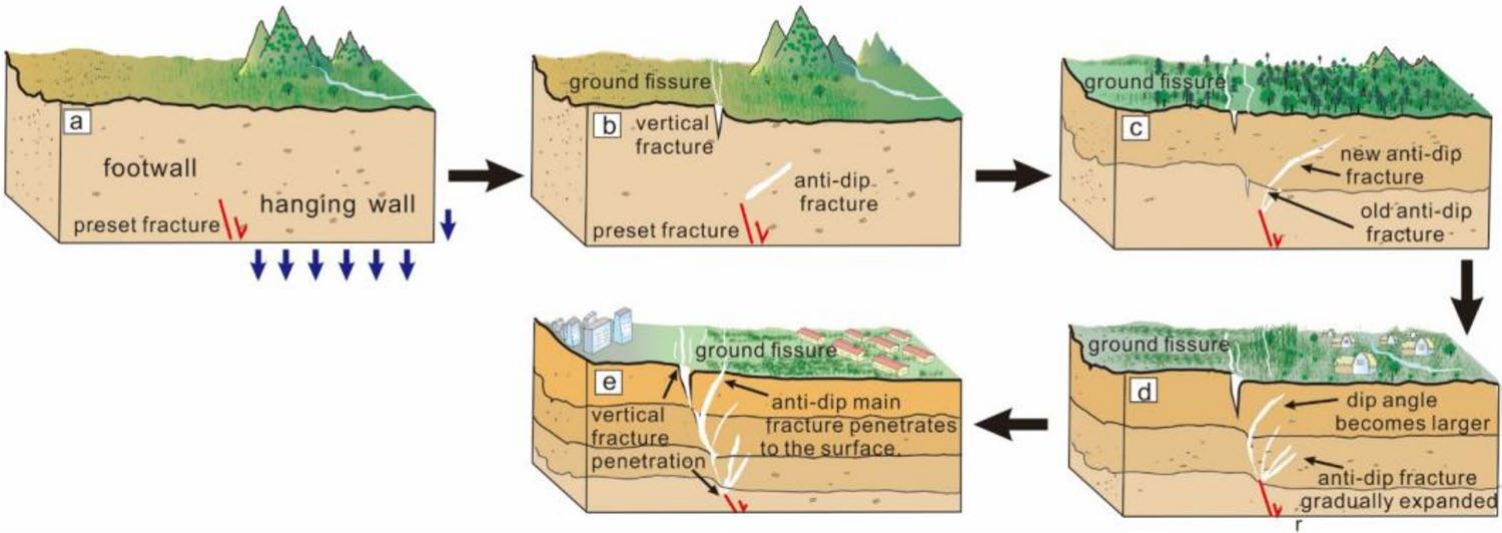
Fracture propagation stage of syn-depositional ground fissures.

Therefore, we divided fracture propagation stages of syn-depositional ground fissures (Fig. 12). In phase a, the preexisting fracture began to move. In phase b, near-vertical and anti-dip fractures were observed. In phase c, under sedimentation, new anti-dip fractures appeared at the tip of the old. In phase d, the dip angle of anti-dip fracture increased, and pre-existing fracture tip was continuously subjected to tensile stress. In phase e, the near-vertical fractures gradually penetrated layers at different depths. The primary anti-dip fracture extended to surface. Secondary anti-dip fractures formed at the tip of pre-existing fracture.

### Comparison of ground fracture modeling tests

**Figure 13:**
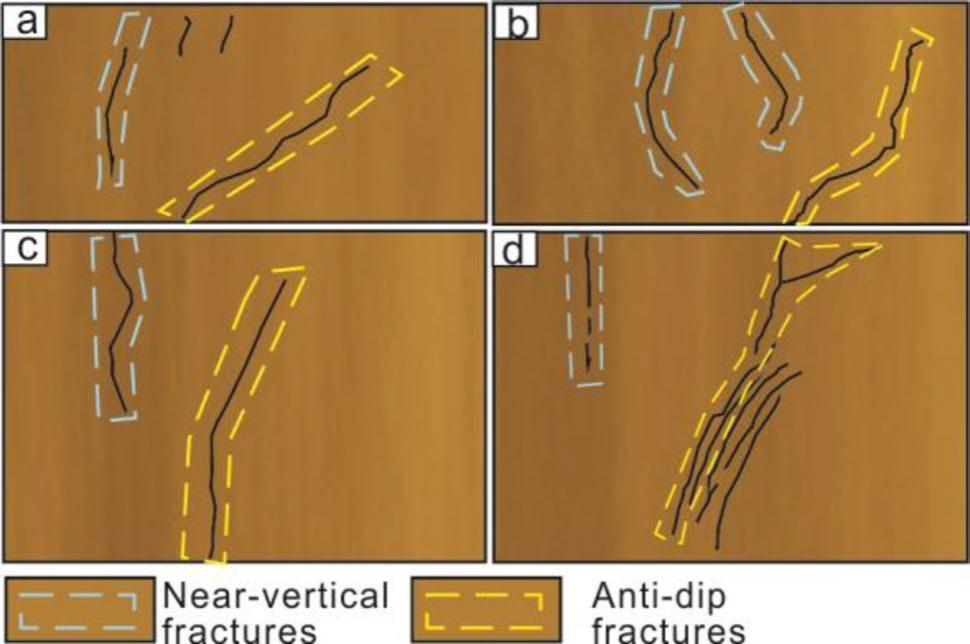
Different patterns of fracture profiles^18,20,37,38^.

The results of the present experiment differ in many ways from those of previous studies of fracture extension in buried ground fissures. The layer profile fracture of the buried ground fissure under the action of tectonic movement consists of two main fractures (anti-dip fracture and near-vertical fracture) (Fig. 13)^18,20,37,38^. The anti-dip fracture is angled and continues to break from the bottom along the angle to the surface. The rupture of the soil shows continuity during the continuous tectonic movement. Secondary fractures in the soil layer disappear in the middle of the formation during propagation. However, the syn-sedimentary ground fissure profile is composed of multiple anticlinal fractures as well as near-vertical fractures. Each stage of the near-vertical fracture will gradually penetrate from the surface towards the bottom of the formation to form a primary fracture. The roots of the anti-dip fracture gradually merge with the main near-vertical fracture.

It is noteworthy that the fracture in the phase 1 of the test showed similar results to the previous test. Starting from Phase 2, the rupture angle of the root of the main anti-dip fracture gradually increased, and many secondary anti-dip fractures began to appear in the hanging wall. This indicates that syn-sedimentary ground fissures were formed by hidden ground fissures in the deep layer through various tectonic movements. These syn-sedimentary ground fissures gradually extend towards the surface from depth. When the fractures propagate into the near-surface layer (a few meters away from the surface), they extend to the surface during surface water infiltration or transitional groundwater pumping by humans. Thus, the development of ground fissures in the layer is characterized by the transformation of buried ground fissures in the deeper layer into syn-sedimentary ground fissures, which then eventually form near-surface buried ground fissures.

## Limitations of model tests

The preparation of the large-scale model layer involves initially watering the soil and then compacting it. The objective is to attain a targeted density and water content in the model layer, ensuring uniformity across different depths. In contrast, the actual soil layer exhibits variations in density and water content with depth and water table levels, leading to changes in cohesive strength^39^. These variations directly impact the mechanical behavior of soil rupturing.

In addition to this, the soils below the water table are saturated soils and their mechanical behavior of fracture expansion is not the same as that of unsaturated soils. The breaking strength of saturated soils decreases under the action of excess pore pressure. Since the cohesion of saturated soils is zero, its fracture process is highly dependent on the angle of internal friction^40^. Therefore, this study cannot accurately model the propagation process of fracture in saturated soils below the water table.

## Conclusion

In this paper, the extension mechanism of syn-sedimentary ground fissure is investigated by large-size physical modeling tests. The main conclusions are as follows:

1. The fracture morphology of the profile gradually changed from a single Y-type to a composite Y-type as the number of layer deposition increased. The main anti-dip fracture changed from nearly 45° straight line to rickety morphology. Its rupture position gradually moved toward the footwall direction.
2. The root of the primary anti-dip fracture at each stage penetrates within the stratigraphic interior to form a near-vertical fracture. The secondary anti-dip fracture of the profile closes completely within the stratigraphic interior.
3. As the number of layer deposits increases, the area of surface deformation gradually moves in the direction of the footwall. Areas of fracture in the layer are classified as bad engineering area. Areas where no fracture occurs, but where soil deformation occurs, are classified as relative engineering. These areas are not conducive to construction.
4. The propagation of syn-sedimentary ground fissures can be categorized into five stages: pre-existing fractures became active; fractures appeared in the layer; syn-sedimentation affected the extension of old fractures; the anti-dip fracture dip angle gradually increased; near-vertical fractures gradually developed towards the depth.

## Data Availability

All data generated or analysed during this study are included in this published article (and its Supplementary Information files).
